# The Beneficence of Homœopathy

**Published:** 1892-07

**Authors:** Rufus Choate

**Affiliations:** Washington, D. C.


### THE BENEFICENCE OF HOMŒOPATHY.

EDITOR OF THE HOMŒOPATHIC PHYSICIAN:

You continue to drive the truth of Homœopathy home, and manfully hold to establishing the purity of the science! Success to you! I can easily see why Homœopathy does not have the loyal support of its professors generally. To obtain a knowledge of her bounties, one must woo her with a devotedness as true and as deep, as fervent and as steadfast as the love he would give the dearest woman that walks the earth. Our dear science wants no half-way devotee. To him who loves her truly, and would give his life rather than drag her down, she comes with riches and sweetest blessings. In the fair days of success, she smilingly blesses him ; in the dark night of sorrow and approaching death she courageously upholds his hands, makes steadfast his determination, and calmly leads from gloom to the bright light of day and life. To him who knows and thoroughly believes the truths of the science of Homœopathy, there is no wavering, no pick-ups, no make-shifts ! No ! He calmly gives his order, and says, " Peace, be still !" Those who hear the voice patiently wait ; but he who believes but half tries to hide his ignorance in curing, and says, do anything for the good of the patient. *Do only* right ; whether it be for good or evil, *do* the right. Follow where truth guides fearlessly. Oh ! could the world but see and know the wondrous beauties and powers for good that exist in true Homœopathy, it would treat with contempt the mongrelism of many of the professed followers of the science.

Let, then, The Homœpathic Physician continue in the fight for the right, and may its career be as prosperous as I wish it. Yours, fraternally,

Rufus Choate, M. D.

Washington, D. C., February 20th, 1892.

